# Therapeutic Effects of *Scutellaria baicalensis* Georgi Extract and Baicalein on Olfactory Dysfunction and Neurobehavioral Alterations in a Methimazole-Induced Injury Model

**DOI:** 10.3390/life16061037

**Published:** 2026-06-22

**Authors:** Manh Nguyen Dao, Hang Thi Nguyet Pham, Nam Duy Pham, Cuong Viet Vo

**Affiliations:** 1 Vietnam-Russia Tropical Science and Technology Research Center, Ha Noi 11307, Vietnam; daonguyenmanh0209@gmail.com (M.N.D.);; 2 Department of Pharmacology and Biochemistry, National Institute of Medicinal Materials, Ha Noi 11018, Vietnam; 3 Phenikaa School of Medicine and Pharmacy, Phenikaa University, Ha Noi 12114, Vietnam

**Keywords:** olfactory epithelial regeneration, olfactory dysfunction, methimazole-induced injury, baicalein, *Scutellaria baicalensis* Georgi

## Abstract

Background: Olfactory dysfunction is a pathology associated with viral infections, toxic damage, aging, and neurodegenerative diseases. Damage to the olfactory epithelium impairs olfactory function and related neurological behaviors. This study evaluated the restorative effects of *Scutellaria baicalensis* Georgi (SBG) extract and baicalein in a methimazole-induced olfactory dysfunction model. Methods: Olfactory epithelial damage was induced in mice with methimazole, followed by treatment with SBG extract or baicalein. Olfactory and neurobehavioral functions were assessed using odor-finding, novel object recognition (NOR), Morris water maze (MWM), open field (OFT), and elevated plus maze tests (EPM). Histological, immunohistochemical, and *in vitro* analyses were performed to evaluate epithelial regeneration, mature olfactory sensory neurons (OSNs) expressing olfactory marker protein (OMP), and proliferative activity. Results: Methimazole induced severe olfactory epithelial damage, impairing olfactory behavior and reducing learning and memory. Treatment with SBG extract and baicalein significantly improved olfactory and cognitive functions. Histological and immunohistochemical analyses confirmed restoration of epithelial structure and olfactory neurons. *In vitro*, SBG extract increased epithelial cell density and modulated proliferative activity. Conclusions: SBG extract and baicalein promote recovery of olfactory function and improve neurobehavioral outcomes, indicating their potential as therapies for olfactory dysfunction.

## 1. Introduction

Olfactory dysfunction is common in individuals with viral infections, chronic rhinosinusitis, toxic exposures, aging, and neurodegenerative diseases. About 20–25% of the general population experience some degree of smell impairment, with prevalence rising to over 60% in those aged 80 and older [1]. Interest in olfactory dysfunction has grown since the COVID-19 pandemic. Post-viral olfactory loss can persist for months and significantly reduce quality of life [2]. Viral and inflammatory insults disrupt olfactory sensory neurons (OSNs), damage sustentacular (Sus) cells, and impair the regenerative capacity of the olfactory epithelium (OE) [3]. These injuries often lead to persistent anosmia or hyposmia.

The OE is a specialized pseudostratified neuroepithelium composed of Sus cells, immature and mature OSNs, and basal stem cells, which include horizontal basal cells (HBCs) and globose basal cells (GBCs). Notably, the OE is one of the few neural tissues capable of lifelong regeneration [4]. This regenerative capacity relies heavily on the activity of HBCs and GBCs. These progenitor cells continuously differentiate into OSNs and other epithelial cells. This process prevents neurodegeneration and olfactory dysfunction induced by various stimuli, thereby preserving olfactory homeostasis. This regenerative process is tightly regulated by signaling pathways that control basal cell quiescence, proliferation, and differentiation [5].

Under physiological conditions, HBCs generally remain quiescent, serving as a reserve stem cell pool. Upon severe epithelial injury, however, they are activated to drive tissue repair by differentiating into multiple olfactory cell types. Conversely, GBCs act as the primary source of neuroregeneration during both homeostasis and post-injury recovery. GBCs express distinct transcription factors at different developmental stages and possess multilineage differentiation potential; they can give rise to non-neuronal lineages, such as Sus cells and Bowman’s gland/duct cells, or differentiate into immature OSNs. These immature neurons subsequently develop into mature OSNs, which express OMP. Basal cell-derived OSNs replace damaged neurons and maintain epithelial homeostasis [6,7].

The recovery of the OE also involves a significant contribution from Sus cells. As specialized supporting cells, they play a central role in maintaining the structural integrity and functional homeostasis of the OE. In addition to providing structural support, Sus cells participate in detoxification processes and contribute to the regulation of neuronal activity. During homeostasis, Sus cells are predominantly derived from GBCs. Following injury, they can arise from multiple cellular sources, including the differentiation of activated HBCs, GBCs, and multipotent Bowman’s gland cells. Sus cells are among the earliest cell types to emerge after injury and actively support basal cell proliferation and neurodifferentiation [8].

This study aimed to evaluate the effects of SBG extract and baicalein on olfactory epithelial recovery, olfactory function, and neurobehavioral outcomes in a methimazole-induced olfactory epithelial injury model. Olfactory epithelial regeneration was assessed through histological examination, epithelial thickness measurements, and immunohistochemical evaluation of OMP-positive mature OSNs and Ki67-positive proliferating cells. In addition, the effects of SBG extract and baicalein on primary olfactory epithelial cell growth were investigated *in vitro*.

SBG is a traditional medicinal herb that contains high levels of flavonoids, such as baicalin and baicalein. These flavonoids exhibit anti-inflammatory, antioxidant, and neuroprotective properties [9]. Baicalein specifically reduces oxidative stress, suppresses pro-inflammatory signaling, and enhances neuronal survival pathways in models of neurological and inflammatory diseases [10,11]. By modulating these cellular mechanisms, SBG extract and baicalein may facilitate OE regeneration by preserving the epithelial microenvironment and supporting basal cell-mediated repair following injury [12].

The methimazole-induced OE injury model is commonly used to investigate olfactory neurodegeneration and epithelial regeneration. Methimazole selectively triggers apoptotic degeneration of mature OSNs, leading to significant epithelial damage, disruption of olfactory signaling, and loss of olfactory function [6,7,13,14]. Studies have shown that methimazole-induced neuronal loss is associated with mitochondrial cytochrome c release and caspase-3 activation. After injury, OE regeneration occurs through the activation and proliferation of basal stem and progenitor cells. Acute inflammatory responses further influence olfactory neuroregeneration through NF-κB signaling pathways in the OE. Importantly, the methimazole-induced injury model preserves the structural integrity of the lamina propria and basal compartment. This feature makes the model a valuable experimental platform for studying regenerative mechanisms and for assessing therapeutic agents that promote olfactory epithelial repair and functional recovery [15,16].

## 2. Materials and Methods

### 2.1. Materials

Male *Swiss albino* mice (National Institute of Hygiene and Epidemiology, Ha Noi, Vietnam) were acquired at 8 weeks of age. Animals were acclimated in the laboratory animal facility for at least one week before experimentation. Food and water were provided ad libitum. Environmental conditions were maintained at 22 ± 1 °C, 65% humidity, and a 12 h light–dark cycle (lights on: 07:00–19:00). All procedures complied with the guidelines of the Committee for the Ethical Use of Experimental Animals, Vietnam–Russia Tropical Science and Technology Research Center, Ha Noi, Vietnam.

SBG roots collected from Bac Ha, Vietnam, were dried to a moisture content below 10% and ground into a fine powder. The powder was extracted with an ethanol–water solvent (EtOH:H_2_O, 8:2, *v*/*v*) at a solvent-to-material ratio of 10:1 (*v*/*w*) at 80 °C for 4 h. The extraction was repeated three times, and the resulting extracts were combined, filtered, and concentrated under reduced pressure. The concentrated extract was spray-dried to yield a dry powder (yield: 25 ± 5% relative to the dried raw material).

For extract standardization, the concentrations of the two major flavonoids, baicalin and baicalein, were determined by high-performance liquid chromatography at the National Institute of Functional Foods, Ha Noi, Vietnam. Quantitative analysis revealed that baicalin and baicalein constituted 21.85% and 3.65% of the dried extract, respectively.

### 2.2. LC–MS/MS Analysis of SBG Extract

Dried SBG extract samples were resuspended in 80% methanol (2 mg/mL), vortexed, and centrifuged at 13,000 rpm for 15 min. The clear supernatant was transferred to sample vials. LC-MS/MS in positive-ion mode was performed on a Bruker Impact II instrument coupled to a Waters UPLC system. The mass detection range was *m*/*z* 100–1500. External mass calibration was performed using sodium formate, introduced at the end of each analytical run [17]. Raw data were processed using Bruker MetaboScape software (version 5.0, Bruker Daltonics, Bremen, Germany). Feature detection was based on retention time and *m*/*z* pairs, followed by molecular feature extraction and alignment. Tentative identification was performed by matching MS/MS fragmentation spectra against several spectral libraries, including the MetaSci library, the MS-DIAL metabolomics MSP spectral library (version 15), NIST20, and an in-house database. Identification was further supported by comparison with exact mass data from the literature.

### 2.3. Methimazole Administration

To induce olfactory epithelial injury, mice received a single intraperitoneal injection of methimazole (50 mg/kg; Sigma-Aldrich, St. Louis, MO, USA) dissolved in 0.9% NaCl on day 0. Methimazole has been shown to induce apoptosis in olfactory neurons, leading to degeneration of the OE, and is therefore widely used in rodent models of olfactory epithelial injury. Previous studies have shown that methimazole at doses of 50, 75, and 100 mg/kg induces olfactory epithelial injury. However, the higher doses (75 and 100 mg/kg) cause extensive epithelial ablation and near-complete loss of the OE. Therefore, a dose of 50 mg/kg was selected to induce injury while preserving sufficient regenerative potential for evaluating treatment-induced recovery [14,18,19].

### 2.4. In Vivo Study

Mice were randomly assigned to experimental groups. The control group (*n* = 12) received no methimazole and was administered distilled water. Methimazole-treated groups included a pathology group (distilled water, *n* = 12), SBG-treated groups (100 and 200 mg/kg, *n* = 11 per group), baicalein-treated groups (20 and 40 mg/kg; Sigma-Aldrich, *n* = 11 per group), and a reference treatment group treated with donepezil (1.5 mg/kg; Sigma-Aldrich, *n* = 11).

The doses of SBG extract and baicalein were determined based on previous studies demonstrating their neuroprotective and cognitive-enhancing effects in experimental models of neuronal injury and memory impairment. SBG extract has been reported to be effective at doses of 50 and 100 mg/kg [20], whereas baicalein has shown significant pharmacological activity at 20 and 40 mg/kg. Accordingly, to evaluate their potential therapeutic effects on olfactory epithelial injury and subsequent dysfunction, doses of 100 and 200 mg/kg were selected for the SBG extract, and 20 and 40 mg/kg were selected for bai-calein [21].

Donepezil was selected as a reference treatment based on previous evidence demonstrating both neuroprotective and olfactory function-enhancing effects [22]. Takahashi et al. showed that donepezil (1.5 mg/kg) ameliorated zinc sulfate-induced olfactory dysfunction in mice by modulating neurogenesis, suppressing α-synuclein aggregation in the olfactory bulb, and enhancing autophagy. Accordingly, donepezil was employed as a pharmacological reference control for olfactory functional recovery and related neurobehavioral outcomes in the present study [23].

Methimazole was used to induce olfactory epithelial injury as described above. Bai-calein (Sigma-Aldrich) and SBG were dissolved in 0.5% carboxymethyl cellulose. Behavioral testing was conducted on day 7 after methimazole-induced injury.

### 2.5. Odor-Finding Test

**Figure 1:**
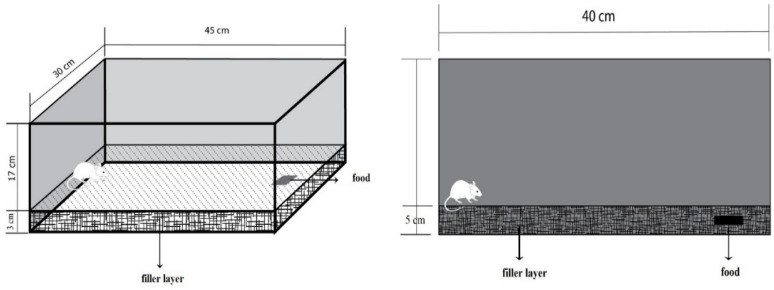
Odor-finding test model.

**Figure 2:**
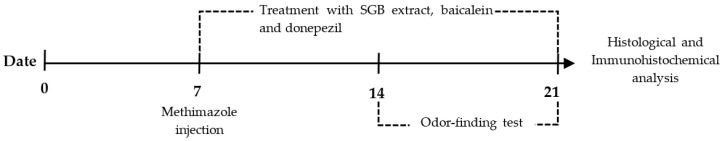
Experimental timeline of olfactory function assessment and histological analyses.

The test was conducted in a wooden box (40 cm × 20 cm × 20 cm) containing a 5 cm layer of bedding. A filter paper (4 cm × 4 cm) impregnated with peanut butter (1.2 g/mL) was used as the odor stimulus (Figure 1). The filter paper was buried beneath the bedding at the center of the target zone, positioned 5 cm from the wall. The test consisted of two phases. During the training phase, mice were allowed to freely explore the arena for 10 min to locate the food and habituate to the environment. This procedure was repeated three times. During the test phase, mice were allowed to freely explore the arena and search for the food within 180 s. Animal movement was recorded using a camera and analyzed with ANY-maze (Stoelting, Wood Dale, IL, USA). If a mouse failed to locate the food within 180 s, the latency was recorded as 180 s. The test was performed on day 7 after methimazole administration (Figure 2) [24,25].

### 2.6. Assessment of Learning and Memory

#### 2.6.1. NOR Test

The test was conducted in a box (40 cm × 40 cm × 50 cm). During the training phase, mice were allowed to explore two identical objects for 5 min. In the test phase, one object was replaced with a novel object of a different shape. Mice were allowed to explore both objects for 5 min. Behavior was recorded using a camera and analyzed using ANY-maze [26].

#### 2.6.2. MWM Test

Spatial learning and memory were assessed in a circular pool (diameter 1.1 m, height 30 cm). A platform was fixed in one quadrant, and distinct visual cues were placed around the pool to provide spatial orientation. On day 1, the platform was positioned 1 cm above the water surface to allow habituation. From days 2 to 6, the platform was submerged 1 cm below the water surface; mice performed four trials per day, each starting from a different quadrant. If a mouse failed to locate the platform within 60 s, the mouse was guided to the platform and allowed to remain there for 20 s. On day 7, a probe trial was conducted without the platform, with mice allowed to swim freely for 60 s. Behavior was recorded using a camera and analyzed using ANY-maze [27,28].

### 2.7. Anxiety Behavior

#### 2.7.1. OFT

The test was conducted in a black-painted wooden box (40 cm × 40 cm × 60 cm). Mice were allowed to freely explore the box for 6 min. Behavior was recorded using a camera and analyzed with ANY-maze (Stoelting, USA), excluding data from the first min of exploration [29].

#### 2.7.2. EPM Test

The test was performed using an elevated plus-shaped maze consisting of two open arms, two closed arms, and a central platform, raised 50 cm above the floor (arm width: 8 cm; length: 25 cm). Mice were placed at the center of the maze and allowed to explore for 5 min. Behavior was recorded with a digital camera and analyzed using ANY-maze [30].

**Figure 3:**
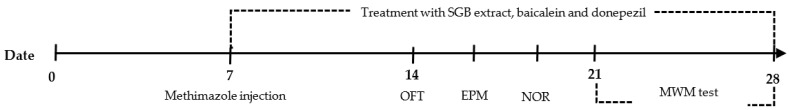
Experimental timeline of neurobehavioral assessments.

A schematic overview of the experimental timeline for the OFT, EPM, NOR, and MWM tests is presented in Figure 3.

### 2.8. Cell Culture

**Figure 4:**
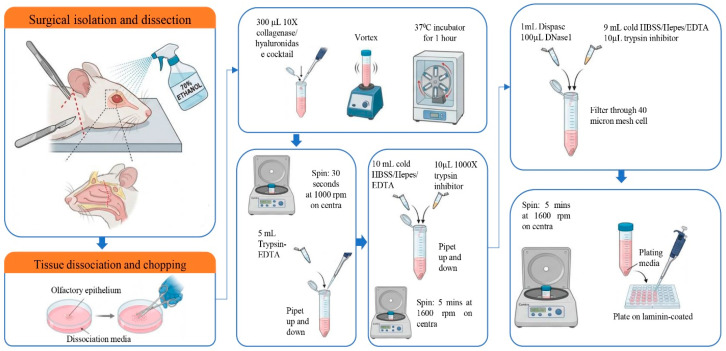
Primary basal cells culture protocol.

The OE was isolated from the nasal septum of adult *Swiss albino* mice under sterile conditions. After surface sterilization with 70% ethanol, the heads were dissected, the turbinates were removed, and the OE region was carefully separated. The tissue was then minced and transferred into 3 mL of dissociation medium. The dissociation medium contained PneumaCult-Ex, Gem21 Neuroplex Supplement, N2 Neuroplex Supplement, penicillin/streptomycin, and ROCK inhibitor Y27632. Samples were minced further, treated with collagenase/hyaluronidase (Stemcell Technologies (Vancouver, BC, Canada), #07912), vortexed, and incubated for 60 min at 37 °C. The suspension was centrifuged at 1000 rpm for 30 s, and the supernatant was removed. The cell pellet was treated with 0.25% trypsin–EDTA (Stemcell Technologies, #07901). The mixture was neutralized with cold HBSS containing trypsin inhibitor. After centrifugation at 1600 rpm for 5 min, the pellet was further digested with dispase (5 mg/mL; Stemcell Technologies, #07913) and DNase I (1 mg/mL; Stemcell Technologies, #07900). This step generated a single-cell suspension. The suspension was filtered through a 40 µm mesh filter and centrifuged again. Cells were resuspended in plating medium (PneumaCult-Ex, Gem21 Neuroplex Supplement, N2 Neuroplex Supplement, ROCK inhibitor Y27632, α-TGF, DMH1, and A83-01) and seeded onto laminin-coated culture dishes, followed by incubation at 37 °C. After 24 h, the plating medium was replaced with maintenance medium (PneumaCult-Ex, Gem21 Neuroplex Supplement, N2 Neuroplex Supplement, α-TGF, DMH1, A83-01, and GlutaMAX), and the medium was refreshed every 48 h until passaging. After 14 days of culture, cells were harvested with Accutase, centrifuged, and resuspended in 1 mL plating medium in Falcon tubes. To determine cell density, a 10 µL aliquot of the suspension was counted using a hemocytometer. The prepared cell suspension was seeded onto coverslips (50 µL per coverslip) at about 4000–5000 cells per coverslip and kept for 4 more days to allow attachment and stabilization. Once cells had stabilized, they were treated with SBG extract and baicalein (Sigma-Aldrich) at concentrations of 125 and 625 µg/mL, respectively. After this treatment, the samples were incubated for 48 h before immunohistochemical staining [31]. A schematic overview of the primary basal cell isolation and culture protocol is presented in Figure 4.

### 2.9. Histological and Immunohistochemical Analysis

#### 2.9.1. *In Vivo*

Mice were anesthetized with chloral hydrate (50 mg/kg). After induction of complete anesthesia, perfusion was performed, followed by decalcification, fixation in 4% paraformaldehyde, paraffin embedding, and sectioning. Samples were sectioned at a level located 1 mm anterior to the olfactory bulb, with a thickness of 5 µm. Nasal sections were deparaffinized in xylene and rehydrated through graded ethanol.

One section was stained with hematoxylin and eosin (H&E) and used to measure epithelial thickness using an inverted phase-contrast fluorescence microscope (Olympus IX73, Olympus Corporation, Tokyo, Japan, 10X objective). Measurements were taken at six different locations per nasal section along both the right and left septum.

Another section was subjected to immunohistochemistry using OMP (goat polyclonal antibody, 1:200 dilution, Thermo Fisher Scientific, Waltham, MA, USA) and Ki67 (rabbit monoclonal antibody, 1:400 dilution, Thermo Fisher Scientific). OMP is a 19 kDa intracellular protein abundantly expressed in mature olfactory receptor neurons, but absent in neural progenitor cells; OMP-positive cells are primarily located in the middle layer of the OE [32]. Ki67 is a well-established marker of cell proliferation, expressed during all active phases of the cell cycle [33], with positive cells typically localized in the basal layer of the OE. Sections were deparaffinized and rehydrated, then antigen-retrieved in citrate buffer at 95 °C for 10 min. Sections were blocked with normal donkey serum in phosphate-buffered saline (PBS) and incubated overnight with primary antibodies. After washing with PBS, sections were incubated with the secondary antibodies for 30 min, washed with PBS, and counterstained with DAPI.

Cell counting was performed using ImageJ software (version 1.54f, National Institutes of Health, Bethesda, MD, USA) by a single blinded evaluator. Quantification was conducted on 100 µm epithelial segments, starting from the dorsal recess and extending ventrally along the epithelium.

#### 2.9.2. *In Vitro*

Cells cultured on coverslips were washed with PBS and fixed in 10% buffered phosphate formalin for 15 min at room temperature. Samples were washed and permeabilized with ice-cold methanol (−20 °C), followed by PBS washes. After blocking, samples were incubated with a primary Ki67 antibody (rabbit monoclonal, 1:400 dilution, Thermo Fisher Scientific) for 4 h at room temperature. Nuclei were counterstained with DAPI before imaging.

Slides were imaged using a Zeiss fluorescence microscope (Carl Zeiss Microscopy GmbH, Jena, Germany). Image analysis was performed using ImageJ (NIH, Bethesda, MD, USA). For each sample, fixed-size regions of interest (ROIs, 0.636 mm^2^) were consistently defined across all images, selected from areas with uniform cell distribution while avoiding overlapping regions, folds, or background artifacts. Cell density was expressed as cells/mm^2^.

### 2.10. Statistical Analysis

Data were analyzed using GraphPad Prism software (version 11.0.0, GraphPad Software, San Diego, CA, USA) and are presented as mean ± SEM. Comparisons among multiple groups were performed using one-way ANOVA followed by Tukey’s post hoc tests. Repeated-measures data from the MWM acquisition phase were analyzed using two-way repeated-measures ANOVA followed by Tukey’s post hoc test. Nonparametric data were analyzed using the Kruskal–Wallis test followed by Dunn’s multiple comparisons test. A *p*-value < 0.05 was considered statistically significant.

## 3. Results

### 3.1. Chromatographic Fingerprinting Profiles of SBG Extract

**Figure 5:**
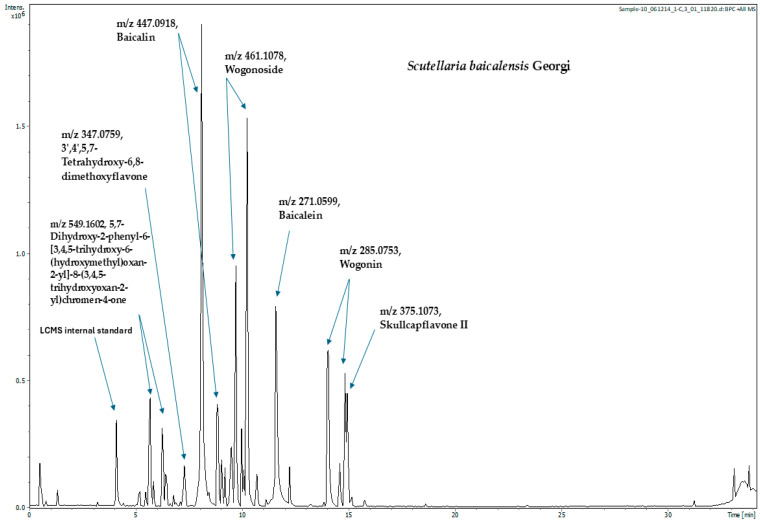
LC–MS base peak chromatogram of SBG extract.

The LC–MS/MS chromatographic analysis of SBG extract revealed a chemically complex composition, primarily consisting of flavonoid glycosides and flavone aglycones (Figure 5). The majority of significant peaks occurred within the 5–15 min retention time range, suggesting that the extract predominantly contains compounds of moderate polarity. This chromatographic pattern aligns with established phytochemical profiles of SBG, in which flavonoids are the principal bioactive components. Baicalin was identified as the dominant constituent among the detected compounds, as indicated by its molecular ion at *m*/*z* ~447 ([M + H]^+^) and pronounced chromatographic intensity. Baicalin is widely recognized as the principal flavone glucuronide in SBG and is frequently cited as the herb’s primary marker compound. Additional characteristic flavonoids, including wogonoside, baicalein, wogonin, and skullcapflavone derivatives, were tentatively identified based on their molecular ions and retention times (Table 1).

The chromatogram revealed the presence of both glycosylated and aglycone forms of flavonoids. Glycosylated compounds, such as baicalin and wogonoside, eluted earlier due to their higher polarity. In contrast, aglycones such as baicalein and wogonin exhibited longer retention times due to their lower polarity and greater hydrophobicity. LC–MS/MS profile confirmed that the extract retained the characteristic flavonoid-rich composition of SBG [34,35].

### 3.2. In Vivo Studies

#### 3.2.1. Olfactory Function Recovery

**Figure 6:**
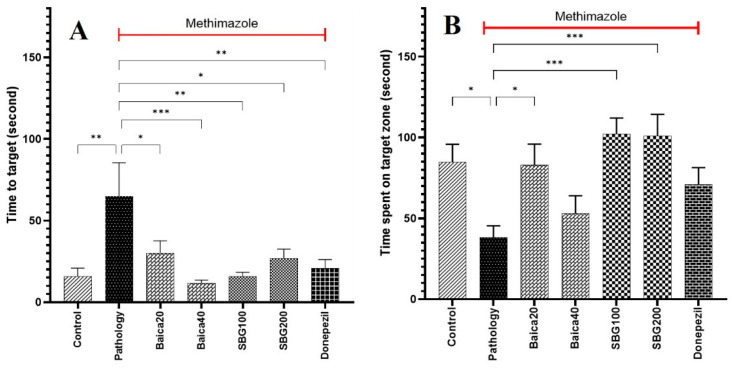
Olfactory function recovery in the odor-finding test. (**A**) Latency to target (maximum 180 s), animals that failed to locate the target were assigned a maximum latency of 180 s. (**B**) Time spent in the target zone. Data are presented as mean ± SEM (*n* = 11–12) with individual values shown. The pathology group showed impaired performance, with increased latency and reduced exploration of the target zone. Statistical significance was determined using one-way ANOVA followed by Tukey’s post hoc test (* *p* < 0.05, ** *p* < 0.01, *** *p* < 0.001 vs. pathology group).

In the odor-finding test, the pathology group (methimazole injection only, distilled water intake) showed a significant decrease in olfactory function, increased latency to reach the target, and reduced time spent in the target zone (Figure 6). These findings suggest that methimazole-induced olfactory epithelial damage impairs odor detection and disrupts olfactory signal processing.

Groups treated with baicalein and SBG demonstrated statistically significant improvements in both measured indicators. A reduction in latency to the target and an increase in time spent in the target zone suggest restoration of olfactory function. Restoration of olfactory function is closely associated with the recovery of the OE, which detects odor molecules.

#### 3.2.2. Effects of SBG Extract and Baicalein on Learning and Memory Performance

**Figure 7:**
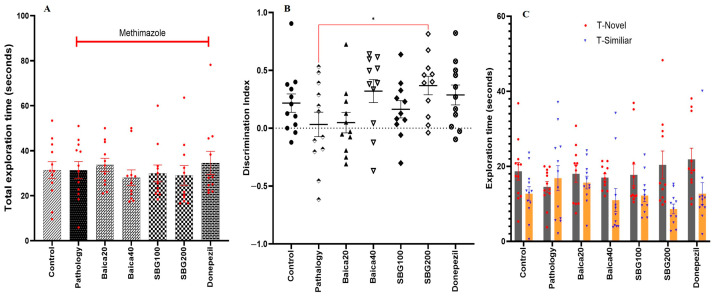
Effects of SBG extract and baicalein in the NOR test. (**A**) Total exploration time during the test phase. (**B**) Discrimination index; (**C**) exploration time in the NOR test. Data are presented as mean ± SEM with individual data points, * *p* < 0.05 vs. pathology group.

**Figure 8:**
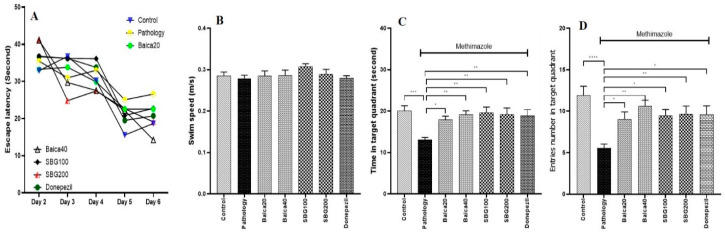
Effects of treatments on spatial learning and memory in the MWM test. (**A**) Escape latency during the acquisition phase (days 2–6). (**B**) Swim speed. (**C**) Time spent in the target quadrant during the probe trial (day 7). (**D**) Number of platform crossings during the probe trial. Data are presented as mean ± SEM. Escape latency data were analyzed using two-way repeated-measures ANOVA followed by Tukey’s post hoc test. Other parameters were analyzed using one-way ANOVA followed by Tukey’s post hoc test. * *p* < 0.05, ** *p* < 0.01, *** *p* < 0.001, **** *p* < 0.0001 vs. pathology group.

Memory and learning impairments in the pathological group were clearly demonstrated in the MWM test, as evidenced by reduced time spent in the target quadrant and fewer platform crossings compared with the control group (*p* < 0.05). The NOR test (Figure 7) also suggested impaired memory and learning performance. However, the differences observed between the pathological and control groups were modest and failed to reach statistical significance (*p* > 0.05). Swimming speed was similar across groups (Figure 8B), indicating that physical condition did not influence the MWM model. Methimazole-induced olfactory epithelial damage disrupts olfactory signaling and reduces neurotransmission within neural circuits involved in learning and memory. OSNs connect directly to the olfactory bulb and transmit signals to the limbic system, including the amygdala and hippocampus, which play crucial roles in memory and emotions. Therefore, damage to the OE not only impairs the ability to recognize smells but also directly affects learning and memory [36].

The groups treated with baicalein and SBG both showed significant improvements in learning and memory, as evidenced by increased time spent in the target quadrant and number of platform crossings in the MWM test. These improvements may not simply be the result of olfactory restoration but also reflect the direct neuroprotective and neuromodulatory effects of these compounds on the central nervous system.

#### 3.2.3. Effects of SBG Extract and Baicalein on Anxiety-like Behavior

**Figure 9:**
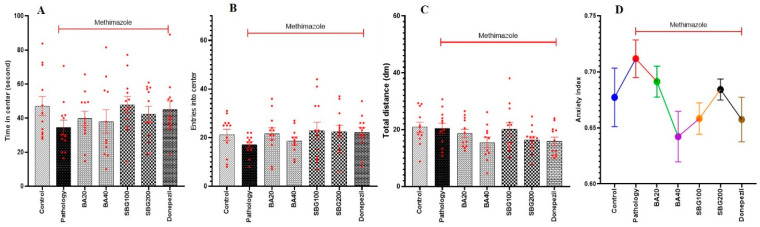
Effects of SBG extract and baicalein on the OFT. (**A**) Time in center, (**B**) entries into center, (**C**) total distance, and (**D**) anxiety index. Data are presented as mean ± SEM (*n* = 11–12), with individual data points shown. One-way ANOVA followed by Tukey’s post hoc test; ns, not significant.

**Figure 10:**
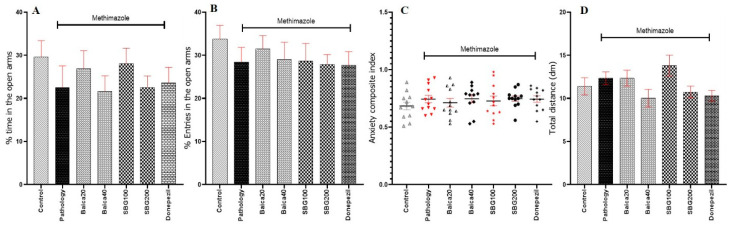
Effects of SBG extract and baicalein on anxiety-like behavior in the EPM test. (**A**) Percentage of time spent in the open arms; (**B**) Percentage of open arm entries; (**C**) Anxiety composite index calculated from open arm time and entries; (**D**) Total distance traveled as an indicator of locomotor activity. Data are expressed as mean ± SEM with individual values shown.

In this study, the OFT and EMP tests did not show significant differences among study groups in exploration indices. Groups treated with baicalein and SBG tended to explore the central area (OFT) and open arms (EPM) more than the pathological group; however, these differences did not reach statistical significance. From these results, it can be preliminarily concluded that methimazole-induced olfactory epithelial damage does not significantly impact anxiety- and depression-like behaviors (Figure 9 and Figure 10). Methimazole primarily damages the OE, not directly affecting central nervous system structures involved in mood regulation [14,16]. Therefore, the observed changes in anxiety-like behavior may be secondary to olfactory functional recovery rather than direct regulation of limbic system activity.

Although not statistically significant, the positive behavioral improvements may be partly explained by the antioxidant and neuroprotective properties of baicalein and SBG, which are thought to regulate biological pathways involved in anxiety disorders. However, further studies using specialized anxiety models and longer observation periods are needed to determine these effects definitively.

### 3.3. Immunohistochemistry and Thickness of OE

Histological analysis revealed that methimazole caused significant damage to the olfactory epithelial structure, significantly reducing epithelial thickness and damaging olfactory neurons. This damage reduced olfactory-related neural signals to the brain, thereby impairing olfactory function and potentially leading to the complete loss of the sense of smell.

**Figure 11:**
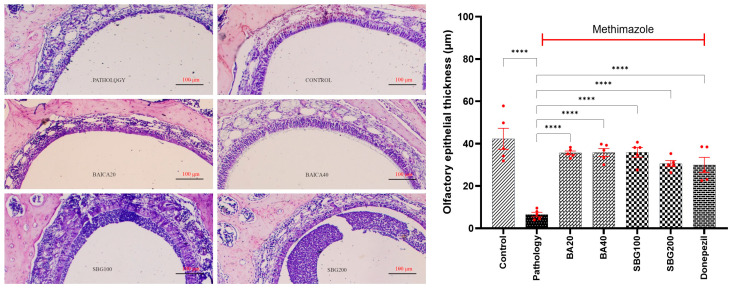
Effects of treatments on olfactory epithelial thickness. Data are presented as mean ± SEM (*n* = 5). Statistical significance was determined using one-way ANOVA followed by Tukey’s post hoc test (**** *p* < 0.0001 vs. pathology group).

The baicalein and SBG treatment groups showed marked recovery of olfactory epithelial structure, with increased epithelial cell types and a significant increase in epithelial thickness compared with the pathological group (Figure 11). Since the OE is one of the few neurogenic tissues capable of regeneration, the increase in epithelial thickness is considered a direct indicator of structural recovery.

**Figure 12:**
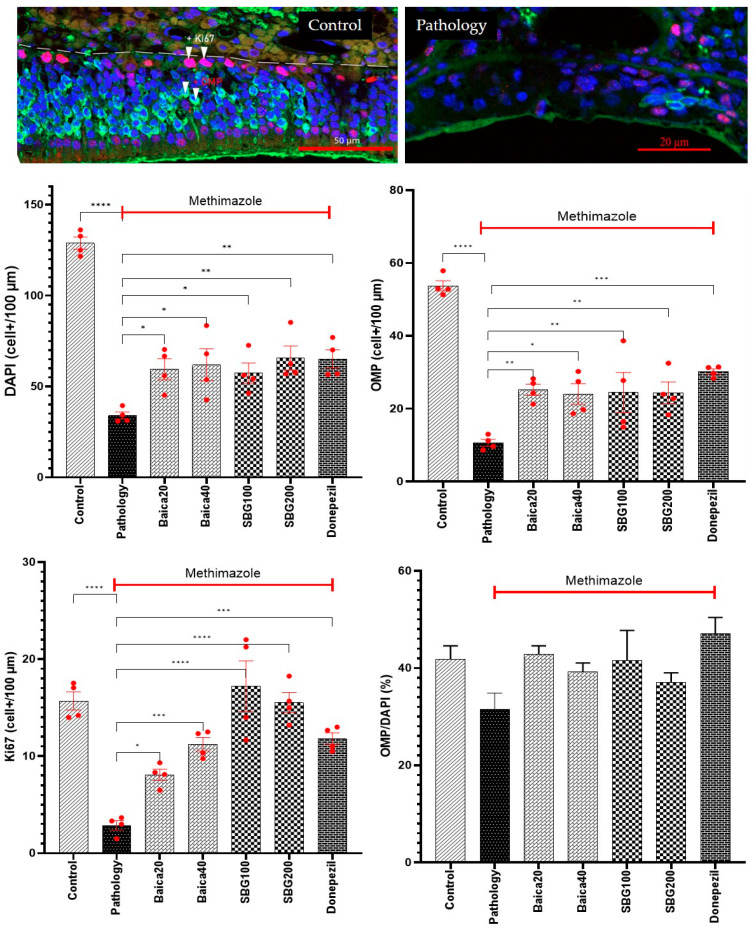
Epithelial cell regeneration after treatment with SBG extract and baicalein. Methimazole significantly reduced DAPI-positive cell density, OMP-positive mature olfactory neurons, and Ki67-positive proliferating cells compared to the control group, indicating severe epithelial damage and impaired regeneration (**** *p* < 0.0001). Treatment with baicalein at 20 mg/kg (Baica20) and 40 mg/kg (Baica40), SBG extract at 100 mg/kg (SBG100) and 200 mg/kg (SBG200), and donepezil significantly restored epithelial cell density, neuronal marker expression, and proliferative activity relative to the pathology group. Data are presented as mean ± SEM (*n* = 4), with individual data points shown. Statistical significance was determined using one-way ANOVA followed by Tukey’s post hoc test (* *p* < 0.05, ** *p* < 0.01, *** *p* < 0.001, **** *p* < 0.0001).

Histological immunohistochemical staining revealed that methimazole significantly reduced the numbers of OMP- and Ki67-positive cells, reflecting damage to mature olfactory neurons and inhibition of epithelial proliferation (Figure 12). This is consistent with methimazole’s mechanism of damage to the OE, which induces selective apoptosis of olfactory neurons and disrupts epithelial regeneration [37].

**Figure 13:**
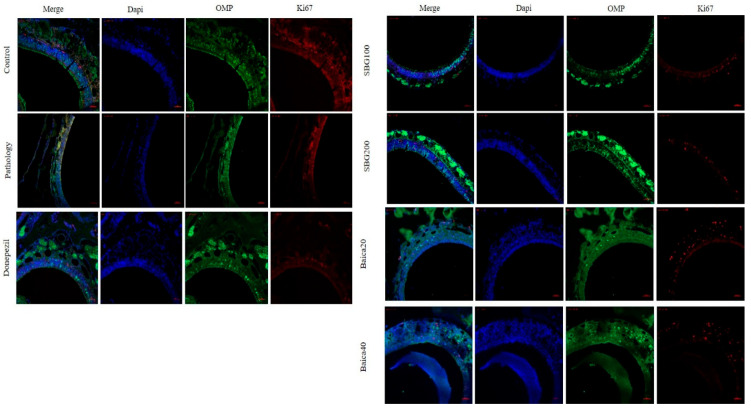
Representative immunofluorescence images of OE following methimazole-induced injury and treatment with SBG extract, baicalein. Scale bar = 100 µm.

Following treatment, the simultaneous increase in numbers of OMP- and Ki67-positive cells in the baicalein- and SBG-treated groups indicated a recovery of epithelial cells (Figure 13). Ki67 is a marker of cells in the active cell cycle, directly reflecting proliferative activity, while OMP is a protein specific to mature olfactory neurons, indicating differentiation and functional recovery. Therefore, the simultaneous restoration of these markers provides evidence of marked cell proliferation and differentiation in the OE after treatment with SBG and baicalein.

### 3.4. Primary Olfactory Epithelial Cell Proliferation

**Figure 14:**
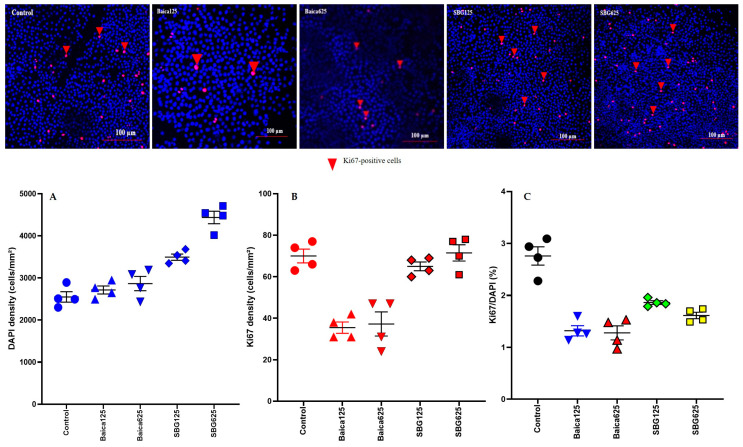
Effects of baicalein and SBG extract on primary olfactory epithelial cell proliferation. (**A**) Total cell density (DAPI), (**B**) Ki67-positive cell density, and (**C**) Proliferation index (% Ki67-positive cells among total DAPI-positive cells). Data are presented as mean ± SEM (*n* = 4 independent experiments), with individual data points shown. Scale bar = 100 µm.

In the *in vitro* experiment, the control group exhibited the highest Ki67/DAPI ratio, indicating the basal proliferative activity of olfactory epithelial cells under optimal culture conditions. Treatment with SBG extract and baicalein significantly increased cell density; however, the number of Ki67-positive cells did not increase proportionally, resulting in a lower Ki67/DAPI ratio than that observed in the control group (Figure 14). These findings suggest that SBG extract and baicalein may primarily promote cell survival, maintenance, and accumulation of olfactory epithelial cells rather than directly enhancing proliferative activity.

Both SBG extract and baicalein significantly increased the density of DAPI-positive cells relative to the control group, with the greatest effect observed in the SBG-treated group at 625 µg/mL. Since DAPI labels nuclear DNA, the number of DAPI-positive cells reflects the total nucleated cell population in the culture, including basal stem cells, progenitor cells, differentiating cells, and mature epithelial cells. In contrast, Ki67 is expressed only in actively cycling cells and is absent in quiescent cells and after mitosis. Therefore, the Ki67/DAPI ratio represents the proportion of proliferating cells within the total cell population rather than an indicator of overall cell viability or differentiation status. The observed increase in DAPI-positive cell density suggests enhanced survival and maintenance of the olfactory epithelial cell population. Although DAPI staining does not distinguish between proliferating and differentiated cells, maintaining epithelial cell density is considered an important feature of olfactory epithelial regeneration and the functional recovery of the olfactory system.

Compared with baicalein, SBG exhibited superior efficacy, as evidenced by a more pronounced increase in both total cell number and Ki67-positive cell count. This enhanced effect may be attributed to synergistic interactions among multiple bioactive compounds present in the SBG extract. Previous studies have demonstrated that SBG contains a diverse array of bioactive flavonoids, including baicalin, baicalein, wogonin, and wogonoside, which are known to regulate signaling pathways involved in cell survival, apoptosis, oxidative stress responses, and tissue regeneration [9,38]. The combined actions of these constituents may therefore produce a greater biological effect than any single compound alone, contributing to the increased density and preservation of olfactory epithelial cells observed in the present study.

## 4. Discussion

This study presents experimental evidence that methimazole at 50 mg/kg induces structural damage to the OE and olfactory receptor neurons. This results in impaired olfactory function, reduced learning capacity, and reduced cognitive performance in vivo. These results are consistent with studies on the effects of methimazole as a selective olfactory toxin in rodents. Although methimazole itself is not inherently toxic, its metabolism by cytochrome P450 enzymes generates reactive metabolites. These enzymes are highly expressed in supporting cells and Bowman’s glands. The metabolites induce chemotoxicity, oxidative stress, and apoptosis in the olfactory mucosa. Apoptotic death of olfactory receptor neurons occurs when cytochrome c is released from mitochondria into the cytoplasm. This activation of caspase-3 initiates cell destruction [16,39]. Consequently, the loss of mature OSNs disrupts olfactory signal transmission and impairs odor perception.

Unlike other sensory systems, the olfactory sensory system transmits signals directly to the limbic system, bypassing the thalamus. The limbic system, which is closely associated with memory and emotional processing, includes the hippocampus, which consolidates short-term memories into long-term storage, and the amygdala, which assigns emotional significance to memories. This distinctive neuroanatomical connection accounts for the strong emotional responses frequently elicited by familiar odors. Disruption of olfactory signal transmission to the limbic system may impair neural information-processing networks in olfactory-related brain regions, thereby contributing to deficits in learning and memory [40].

In the present study, the NOR test revealed a trend toward improved recognition memory in the treatment groups, as reflected by increased exploration of the novel object. However, these differences (*p* > 0.05) were relatively modest and less pronounced than those observed in the MWM test. In contrast, the MWM results demonstrated significant differences among groups (*p* < 0.05), indicating that methimazole-induced olfactory epithelial injury was associated with impaired spatial learning and memory. Treatment with SBG extract and baicalein significantly improved learning and memory performance in the MWM test.

The structural and functional recovery of the OE may facilitate the restoration of sensory signaling from the peripheral olfactory system to limbic brain regions, particularly the hippocampus and amygdala, which are critically involved in memory formation and emotional processing. Restoration of olfactory sensory input may help maintain neuronal activity and cognitive function within these brain regions. Therefore, it contributes to the improvement in learning and memory. However, the present findings do not allow the conclusion that cognitive improvement was solely attributable to the restoration of olfactory function. Previous studies demonstrated that baicalein and the main flavonoids in SBG, baicalin, wogonin, and wogonoside, exhibited direct neuroprotective effects in the central nervous system by antioxidant, anti-inflammatory, anti-apoptotic, and mitochondrial protective mechanisms. These effects have been associated with enhanced neuronal survival, promotion of synaptic plasticity, and improvements in cognitive function across diverse experimental models [10,11,41,42,43,44]. Therefore, the improvements in learning and memory observed in the present study may result from a combination of mechanisms, including both the restoration of olfactory function and the direct neuroprotective actions of SBG and its bioactive constituents on the brain.

In contrast, no significant differences were observed among the experimental groups in the OFT and EPM (*p* > 0.05), indicating that neither methimazole-induced olfactory epithelial injury nor subsequent treatment significantly affected anxiety- or depression-like behaviors under the conditions employed in this study. These findings suggest that the current model was insufficient to induce detectable emotional disturbances in the behavioral paradigms examined. Although previous studies have reported associations between olfactory dysfunction and affective disorders, including anxiety and depression, several factors may account for the discrepancy between those findings and the present results. First, the olfactory injury induced by methimazole in this study was acute or sub-acute rather than chronic. Second, the OE possesses a remarkable intrinsic regenerative capacity, allowing relatively rapid regeneration following injury. Consequently, the duration and severity of olfactory dysfunction may not have been sufficient to trigger the neurobiological alterations required for the development of measurable emotional disturbances. This interpretation is consistent with previous reports suggesting that long-term olfactory deficits are more strongly associated with anxiety- and depression-related behavioral changes [16,45,46,47].

The restorative effects of SBG extract and baicalein on the OE in this study may be due to the protection of olfactory epithelial cells and restoration of OSNs. They may also enable recovery of neural signal transmission pathways and improve neural network activity in cortical brain regions [48]. Immunohistochemical analysis provided further evidence of methimazole-induced damage to the OE and restorative effects of SBG and baicalein. The pathological group had a simultaneous reduction in OMP- and Ki67-positive cells, reflecting both loss of mature olfactory neurons and impaired proliferative activity. After treatment, increased expression of these two markers indicated activation of epithelial regeneration, including the restoration of mature OSNs and enhanced proliferative activity.

In the *in vitro* study, primary olfactory epithelial cells were cultured in a Pneu-maCult-Ex-based plating medium supplemented with Gem21 NeuroPlex, N2 NeuroPlex, the ROCK inhibitor Y27632, TGF-α, DMH1, and A83-01. This medium was formulated to support epithelial cell viability and progenitor cell expansion via multiple pro-survival and proliferative signaling pathways. Consequently, the baseline proliferative and differentiation activities of these cells were already enhanced before treatment with SBG extract or baicalein. Following exposure to SBG extract and baicalein, a significant increase in cell density was observed within the culture wells, potentially reflecting improved cell survival and the maintenance of the olfactory epithelial cell population [6,49].

The biological properties of SBG further support this interpretation. Previous studies have demonstrated that SBG and its major flavonoid constituents exert antioxidant, anti-apoptotic, mitochondrial-protective, and cytoprotective effects in various cell types [38]. Through these mechanisms, SBG may enhance the survival of both olfactory epithelial progenitor cells and differentiated epithelial cells under *in vitro* culture conditions. Such effects could contribute to the observed increase in overall cell density even in the absence of a proportional increase in the fraction of Ki67-positive cells. These *in vitro* findings should be interpreted as evidence that SBG extract supports the maintenance of the olfactory epithelial cell pool, rather than definitive proof of robust proliferation. However, the current data do not allow for discrimination between enhanced proliferation, reduced apoptosis, and increased differentiation as the predominant underlying mechanism. Further studies employing lineage-specific markers for olfactory progenitor cells, immature neurons, mature neurons, and apoptosis-related proteins are required to clarify the relative contributions of these processes to the observed increase in cell density.

**Figure 15:**
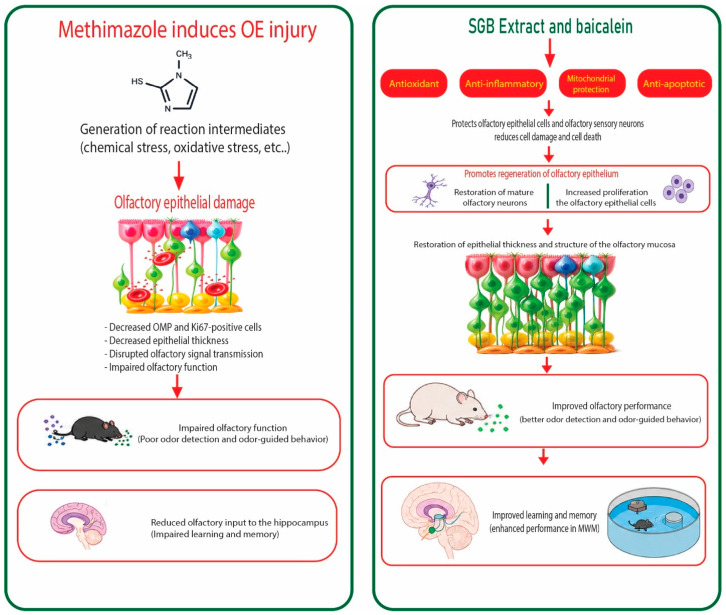
Effects of SBG and Baicalein on a methimazole-induced model of olfactory impairment.

A limitation of this study is that the evaluation of OE regeneration focused solely on epithelial thickness and the expression of OMP and Ki67. Although these markers provide critical insights into OE recovery, mature OSNs, and cell proliferation, they fail to reflect the lineage progression of basal stem cells, progenitor cells, and immature neurons. Therefore, further studies are warranted to investigate the associated molecular mechanisms and signaling pathways, thereby comprehensively elucidating the cellular basis driving the regenerative effects of SBG extract and baicalein. However, the findings support SBG as a promising candidate for treating olfactory dysfunction (Figure 15). Given the increasing number of cases of olfactory dysfunction, the development of multi-targeted natural therapeutic products will offer significant practical value.

## 5. Conclusions

The study provided scientific evidence for the restorative effects of SBG extract and baicalein on olfactory function and memory in a methimazole-induced model of olfactory epithelial damage. This effect was achieved through improved olfactory perception behavior, epithelial structural regeneration, restoration of mature olfactory neuronal populations, and improved spatial memory in the MWM model. *In vitro* experiments indicate that SBG extract and baicalein facilitate olfactory epithelial cell regeneration by modulating cell dynamics and increasing the density of mature cells. These results suggest the therapeutic potential of SBG for olfactory dysfunction and establish a basis for future investigations into molecular mechanisms and clinical applications.

## Figures and Tables

**Table 1:** Tentative identification of major compounds in SBG extract by LC–MS/MS analysis.

Peak	Retention Time (min)	Tentative Compound	Molecular Ion (*m*/*z*)
P1	4.8–5.2	Scutellarein derivative	~463 [M + H]^+^
P2	5.8–6.2	Oroxylin A glucuronide	~461 [M + H]^+^
P3	7.8–8.4	Baicalin	~447 [M + H]^+^
P4	9.2–9.8	Wogonoside	~461 [M + H]^+^
P5	11.3–11.8	Baicalein	~271 [M + H]^+^
P6	13.5–14.0	Wogonin	~285 [M + H]^+^
P7	14.5–15.0	Skullcapflavone II	~375 [M + H]^+^

## Data Availability

Data is contained within the article.

## Abbreviations

The following abbreviations are used in this manuscript:

SBG	*Scutellaria baicalensis* Georgi
OMP	Olfactory Marker Protein
OSN	Olfactory sensory neuron
OE	Olfactory epithelium
GBC	Globose basal cell
HBC	Horizontal basal cell
Sus	Sustentacular
NOR	Novel object recognition
MWM	Morris water maze
OFT	Open field test
EPM	Elevated plus maze
H&E	Hematoxylin and eosin
PBS	Phosphate-buffered saline
